# GABA receptor ameliorates ventilator-induced lung injury in rats by improving alveolar fluid clearance

**DOI:** 10.1186/cc11298

**Published:** 2012-04-05

**Authors:** Narendranath Reddy Chintagari, Lin Liu

**Affiliations:** 1 Lundberg-Kienlen Lung Biology and Toxicology Laboratory, Department of Physiological Sciences, Oklahoma State University, 264 McElroy Hall, Stillwater, OK 74078, USA

## Abstract

**Introduction:**

Mechanical ventilators are increasingly used in critical care units. However, they can cause lung injury, including pulmonary edema. Our previous studies indicated that γ-aminobutyric acid (GABA) receptors are involved in alveolar-fluid homeostasis. The present study investigated the role of GABA receptors in ventilator-induced lung injury.

**Methods:**

Adult female Sprague-Dawley rats were subjected to high-tidal-volume ventilation of 40 ml/kg body weight for 1 hour, and lung injuries were assessed.

**Results:**

High-tidal-volume ventilation resulted in lung injury, as indicated by an increase in total protein in bronchoalveolar fluid, wet-to-dry ratio (indication of pulmonary edema), and Evans Blue dye extravasation (indication of vascular damage). Intratracheal administration of GABA before ventilation significantly reduced the wet-to-dry ratio. Further, histopathologic analysis indicated that GABA reduced ventilator-induced lung injury and apoptosis. GABA-mediated reduction was effectively blocked by the GABA_A_-receptor antagonist, bicuculline. The GABA-mediated effect was not due to the vascular damage, because no differences in Evans Blue dye extravasation were noted. However, the decrease in alveolar fluid clearance by high-tidal-volume ventilation was partly prevented by GABA, which was blocked by bicuculline.

**Conclusions:**

These results suggest that GABA reduces pulmonary edema induced by high-tidal-volume ventilation via its effects on alveolar fluid clearance and apoptosis.

## Introduction

Lung alveoli are lined by a thin layer of fluid that is critical for efficient gas exchange. The fluid balance is maintained by various ion channels expressed on both squamous alveolar epithelial cells type I (AEC I) and cuboidal AECs type II. AEC I express highly selective epithelial Na^+ ^channels (ENaCs), cation-nonselective channels (NSCs), aquaporin 5, and cyclic nucleotide-gated channels. AEC II express ENaC and NSC apically. Na^+^-K^+^ATPase is basolaterally located in both AECs I and AECs II. Both the cell types might mediate alveolar fluid clearance (AFC) because of the expression of ion channels. AFC is mediated by increased Na^+ ^resorption through apically located ENaC and basolateral Na^+^-K^+^ATPase [1]. Conversely, dopamine also increases AFC by enhancing Na^+ ^uptake via the activation of dopaminergic D1-type receptors [2]. Chloride channels play an important role in fluid balance [3,4]. Cl^- ^conductance is mediated by many channels, such as Na^+^-K^+^-2Cl^- ^co-transporter (NKCC), K^+^-Cl^- ^cotransporter (KCC), cystic fibrosis transmembrane conductance regulator (CFTR), and volume- and voltage-gated Cl^- ^channels. These channels are expressed on both AEC I and AEC II. The Cl^- ^diffuses paracellularly and/or through chloride channels, along with Na^+^, to maintain electroneutrality [5-8]. CFTR-mediated Cl^- ^influx results in fluid resorption [9,10]. Water is resorbed by aquaporins expressed on AEC I. Conversely, Cl^- ^effluxes have also been reported [11-14]. Thus, AEC II can mediate the movement of Cl^- ^in both directions.

Our recent studies revealed that UTP regulates Cl^- ^transport through diverse mechanisms. Interestingly, UTP increases Cl^- ^influx in AEC I, but Cl^- ^efflux in AEC II [15]. Thus, AEC I and AEC II appear to mediate Cl^- ^fluxes in different directions.

GABA receptors are multi-subunit Cl^- ^channel receptors and are classified into types A and B, based on subunit composition. GABA_A _receptors are fast-acting ligand-gated ion channels and are mostly inhibitory. However, excitatory receptors have also been reported [16]. GABA_A _can be subdivided into GABA_A-_ρ subtype, which is exclusively composed of ρ (rho) subunits [17]. AEC I and AEC II express many GABA-receptor subunits, which can potentially form functional GABA receptors [18]. GABA receptors are involved in AFC [11]. In adult lungs, glutamic acid decarboxylase, an enzyme involved in GABA synthesis, is localized in AEC II, but not in AEC I, indicating that AEC II is the source of GABA in the distal lung [11]. GABA receptors play an important role in asthma [19-21]. GABA reduces pulmonary adenocarcinoma when hamsters are exposed to nicotine-derived carcinogen. Thus GABA also acts as a potent tumor suppressor [22]. However, its role in alleviating ventilator-induced lung injury is unknown.

Mechanical ventilators are extensively used in critical care units. High-volume ventilation causes epithelial damage, oxidative stress, surfactant inactivation, and pulmonary edema [23-26]. It also increases the expression of pro-inflammatory cytokines [27-29]. Multiple mechanisms of pulmonary edema clearance have been proposed. β-Adrenergic agonists improve the ability of the lungs to clear alveolar fluid in rats after injurious mechanical ventilation [30,31]. This is due to enhanced translocation of the Na^+^-K^+^ATPase enzyme from intracellular compartments to the plasma membrane, rather than an increased expression of the enzyme [30]. Additionally, overexpression of Na^+^-K^+^ATPase improves fluid clearance in various lung-injury models, indicating the potential for gene therapy [32,33]. Losartan, which inhibits angiotensin II, and silvestat, which inhibits neutrophil elastase activities, have also been shown to attenuate lung injury after high-tidal-volume ventilation [34,35]. Chloride channels, particularly CFTR, have been studied for their roles in resolving hydrostatic edema [9]. Similarly, *CFTR *gene transfer increases fluid clearance in rats and mice [36]. However, no studies indicate the role of GABA receptors in resolving pulmonary edema due to inadvertent use of mechanical ventilators. Ways to reduce ventilator-induced lung injury are being extensively studied. The present study attempted to investigate the effects of GABA-receptor modulation on ventilator-induced lung injury.

## Materials and methods

### Reagents

All chemicals and reagents were purchased from Sigma Aldrich (St. Louis, MO, USA) unless stated otherwise. Bicuculline was obtained from MP Biomedicals (Solon, OH, USA). Bovine serum albumin standards and protein assay kits were from BioRad (Hercules, CA, USA).

### Mechanical ventilation

Adult female Sprague-Dawley rats (250 to 300 g) were used for mechanical ventilation. The rats were obtained from Harlan Laboratories (Indianapolis, IN, USA) and were inbred at the Laboratory Animal Research Unit of Oklahoma State University. The animals were randomized with respect to their estrous cycles. Hence it is unknown whether any effects of estrous on the experimental outcomes were a factor. All procedures were approved by the Institutional Animal Care and Use Committee at Oklahoma State University. The rats had free access to food and water *ad libitum *and were maintained on a 12-hour light-dark cycle. The rats were anesthestized with intraperitoneal administration of ketamine (80 mg/kg body weight (BW)) and xylazine (10 mg/kg BW). After anesthesia, the animals underwent tracheotomy. An 18-guage blunted needle was then inserted into the trachea and firmly secured. The rats were ventilated with a small-animal rodent ventilator (CWE Inc, Ardmore, PA, USA) by using the following settings: respiratory rate, 40 breaths/minute; tidal volume, 8 ml/kg BW; inspiratory:expiratory ratio of 1:1; positive end-expiratory pressure (PEEP), 3 cm H_2_O; and FiO_2_, 0.21 for 30 minutes. Later the tidal volume increased slowly to 40 ml/kg BW with a PEEP of 0 cm H_2_O. The animals were ventilated for 30 and 60 minutes. The body temperature of the animals was held constant by placing them on a temperature-controlled (37°C) heating pad. Similar tidal volumes with varying times (40 minutes to 4 hours) have been used in previous studies [30,35,37-41]. A positive PEEP improved lung functioning in humans, whereas it exacerbated functioning in preclinical models [42,43].

For studying the role of GABA receptors in modulating the ventilator-induced lung injury, the animals were ventilated for 15 minutes at a low tidal volume of 8 ml/kg BW with a PEEP of 3 cm H_2_O. The GABA-receptor modulators dissolved in normal saline were then intratracheally instilled slowly over a period of 2 minutes by using a syringe. The volume of the instillate was 1.5 ml/kg BW. The concentrations of GABA and bicuculline (in micromoles) were 500 and 200, respectively. The concentrations of GABA-receptor modulators were based on our previous study. Intratracheal but not systemic administration of GABA affected AFC in adult rats [11], so we instilled GABA intratracheally. Low-tidal-volume ventilation was continued for 15 minutes before subjecting the animals to high-tidal-volume ventilation for 1 hour.

### Analysis of bronchoalveolar lavage

After ventilation, the animals were killed, and the lungs were perfused with saline. A knot was placed at right main bronchus near the tracheal bifurcation. The knot prevented effectively the lavage of the right lungs. The left lungs were lavaged 4 times with 3 ml of normal saline. The lavages from the left lungs were pooled and centrifuged at 170 *g *for 10 minutes. The cell pellet was resuspended in 1 ml saline, and cell counting was done. The cells were also cytospun onto glass slides for differential counting by using Diff Quick stain (Dade Behring, Newark, DE, USA). After staining, at least 200 cells were counted in randomly chosen fields. The numbers of macrophages and neutrophils were expressed as a percentage of total cells. For protein determination, the BAL fluids (BALFs) were made up to equal volume with saline. The protein concentration was determined by using a Bradford assay kit and bovine serum albumin standards (BioRad). The right lungs were used for wet-to-dry ratio and Evans Blue extravasation.

### Wet-to-dry ratio analysis

The wet weight of one of the unlavaged right lobes was recorded and subjected to drying at 60°C for approximately 72 hours. The dry weights were monitored until two successive weights were similar. Later, the wet-to-dry ratio was calculated.

### Histopathology

After high-tidal-volume ventilation, the unlavaged left lungs were fixed by instilling 3 ml of buffered 4% formaldehyde overnight. The fixed tissues were paraffin embedded and processed in three-step sections (that is, sections were made at three different levels in the same lung). The three sections were mounted in one block. Later, 4-μm sections were mounted onto glass slides for further staining. The slides were stained with hematoxylin and eosin.

### TUNEL assay

The lung sections were examined for apoptosis due to ventilator-induced lung injury by using *in situ *cell-death detection kit from Roche Diagnostics (Indianapolis, IN, USA). The kit detects DNA fragmentation in apoptotic cells with the terminal deoxynucleotidyl transferase dUTP nick-end labeling (TUNEL) reaction [44]. Positive cells were detected with the horseradish peroxidase reaction, as described by the manufacturer. Five images (×40 magnification) were captured at each level. Because we had sections at three different levels, 15 images per lung or animal (*n *= 6 to 8) were captured per treatment condition. Care was taken to not include airways and vasculature in the microscopic fields. The numbers of TUNEL-positive cells were counted in all 15 fields, and an average number was calculated. The enumerator was blinded to the experimental conditions.

### Evans Blue dye extravasation

Evans Blue (EB) dye extravasation was done exactly as described before [45]. EB dye was injected into the jugular vein (20 mg/kg BW) 30 minutes before high-tidal-volume ventilation, and the animals were then ventilated as described earlier. The lungs were perfused, and the unlavaged right lung lobes were used for EB dye extraction with formamide. The amount of EB dye was quantified by using EB standards, as described [45].

### Alveolar fluid clearance

Alveolar fluid clearance (AFC) was done as described [11,46]. The ventilation strategy was similar to that described earlier. The animals were ventilated for 15 minutes at 8 ml/kg BW. The body temperature was maintained by placing the animals on a heated pad. After equilibration for 15 minutes, a 20-gauge intravenous catheter was gently inserted into the left lungs. Later, the 5% albumin solution in Ringer lactate (137 m*M *NaCl, 4.67 m*M *KCl, 1.82 m*M *CaCl_2_*2 H_2_O, 1.25 m*M *MgSO_4_*7 H_2_O, 5.55 m*M *dextrose, and 12 m*M *HEPES, pH 7.4 at 37°C) containing 1 mg/ml FITC-albumin, was instilled (1.5 ml/kg BW) into the left lungs. GABA (500 μ*M*) and/or bicuculline (200 μ*M*) was dissolved in the solution containing FITC-albumin. The catheter was gently removed after instillation, and the ventilation was continued for 15 minutes before increasing the tidal volume to 40 ml/kg BW. At the end of ventilation, rats were killed by severing the abdominal aorta. The thoracic cavity was opened, and the lungs were examined to confirm the site of instillation in the left lungs. The FITC-albumin color was noted in the left lungs only. An intravenous catheter (22-gauge) was reinserted, and the instillate was collected again. The instillate was immediately assayed for FITC-albumin concentration by using a spectrofluorometer (HORIBA Jobin Yvon, Edison, NJ, USA). The FITC-albumin concentration was quantified by using FITC-albumin standards. The stock FITC-albumin was also assayed for confirming the change in FITC-albumin concentration before and after ventilation. AFC was calculated as follows: AFC (% decrease) = ((C_initial _- C_final_)/C_initial_) × 100. Our previous study indicated that picrotoxin, a potent GABA-receptor inhibitor, did not affect basal AFC in normal anesthetized animals.

### Statistical analysis

The experiments were done in groups of two, and a Student *t *test was used to compare the differences between treatment groups. A value of *P *< 0.05 was considered significant.

## Results

### Ventilator-induced lung-injury model

The rats were subjected to high-tidal-volume ventilation for 1 hour. After ventilation, the BAL was analyzed for total cells, differential cell counts, and total protein. It should be noted that these cell numbers were obtained from lavage of the left lungs. High tidal volume did not significantly increase the total cell numbers (Table 1). The neutrophils did not increase significantly when compared with those of nonventilated controls after 60 minutes of ventilation. Thus, inflammation was not severe after high-tidal-volume ventilation for 1 hour under our conditions. The amount of total protein (milligrams per milliliter) in BALF was 0.07 ± 0.01 in control animals and increased to 0.15 ± 0.02 after ventilation for 1 hour. The increase in the BALF protein amount indicates lung injury.

**Table 1:** Effect of injurious mechanical ventilation on indices of lung injury

	Number	Total cells (×10^5^)	Protein (mg/ml)	Wet-to-dry ratio	EB dye (μg/g wet tissue)
Control	5	5.6 ± 0.6	0.07 ± 0.01	4.7 ± 0.2	61.5 ± 5.6

30 minutes	4	6.0 ± 0.48	0.13 ± 0.01^a^	6.7 ± 0.1^a^	99.6 ± 8.2^a^

60 minutes	4	6.60 ± 0.8	0.15 ± 0.02^a^	7.5 ± 0.7^a^	133.5 ± 25.7^a^

Values are expressed as mean ± SEM. ^a^*P *< 0.05, versus control.

An increase in wet-to-dry ratio is an indication of fluid accumulation in the lung. The wet-to-dry ratio was 4.7 ± 0.2 in control animals and increased to 6.7 ± 0.2 after ventilation for 30 minutes. The wet-to-dry ratio was also higher after 60 minutes (7.5 ± 0.7) of high-tidal-volume ventilation. Pulmonary edema was evident as early as 30 minutes and persisted up to 60 minutes when compared with that in nonventilated animals (Table 1).

We further determined whether pulmonary edema is due to vascular damage. Rats were injected with EB dye before mechanical ventilation to monitor the extravasation into pulmonary tissues. The amount of EB dye (micrograms per gram wet tissue) in the control lungs was 61.5 ± 6.0 and increased to 99.6 ± 8.3 after ventilation for 30 minutes. The EB dye extravasation doubled (135.5 ± 25.6) at the end of 60 minutes (Table 1).

### Effect of GABA-receptor modulators on indices of ventilator-induced lung injury

These results indicate that high-tidal-volume mechanical ventilation for 1 hour caused pulmonary edema, as shown by an increase in the wet-to-dry ratio. With these conditions, we investigated the role of GABA receptors in modulating ventilator-induced lung injury. Saline-treated animals were used as controls, because GABA was dissolved in saline. We did not find significant differences in lung-injury parameters (total cells, differential counts and protein in BALF, wet-to-dry ratio, and EB dye extravasation) between animals instilled with and without saline.

**Figure 1:**
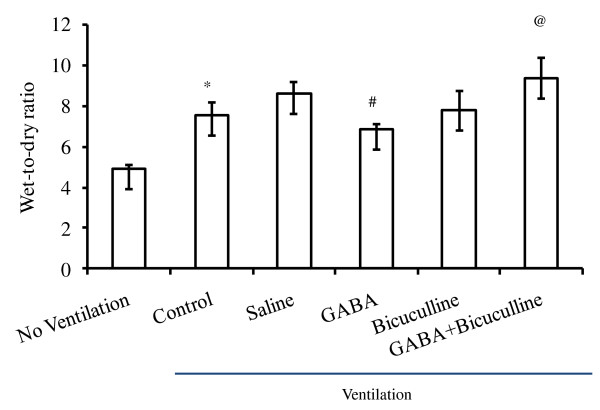
**Effect of γ-aminobutyric acid (GABA)-receptor modulators on ventilator-induced pulmonary edema**. Adult female rats were subjected to a high tidal volume (40 ml/kg BW) ventilation for 60 minutes with and without instillation of GABA (500 μ*M*) and bicuculline (200 μ*M*). The control lungs were instilled with saline. At the end of ventilation, lungs were analyzed for edema formation by measuring the wet-to-dry ratio. Data shown are the mean ± SEM (*n *= 4 to 6 per group). *P < 0.05 versus nonventilated animals; ^#^*P *< 0.05 versus saline; and ^@^*P *< 0.05 versus GABA.

No differences in wet-to-dry ratio were found between the animals ventilated with and without saline instillation (8.6 ± 0.5 versus 7.5 ± 0.6). However, instillation of GABA significantly reduced the wet-to-dry ratio to 6.9 ± 0.2. Bicuculline is a specific antagonist of GABA_A _receptors and does not inhibit GABA_B _and GABA_C _receptors [47]. Instillation of bicuculline alone did not affect the wet-to-dry ratio (7.9 ± 0.9). However, a GABA-mediated decrease in the wet-to-dry ratio was effectively inhibited by bicuculline (9.3 ± 1.0). Thus GABA-mediated effects were due to GABA_A _receptors (Figure 1).

### Effect of GABA on ventilator-induced lung injury

**Figure 2:**
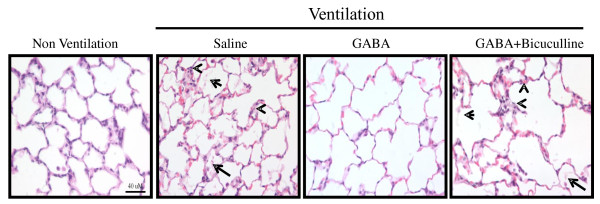
**Effect of γ-aminobutyric acid (GABA) on lung injury after high-tidal-volume ventilation**. Adult female rats were anesthetized and intratracheally instilled with GABA (500 μ*M*) and bicuculline (200 μ*M*). Later, the animals were ventilated at a high tidal volume of 40 ml/kg BW for 60 minutes. At the end of ventilation, the left lungs were fixed for histopathology. Shown are the representative photomicrographs (×400) of lung tissue stained with hematoxylin and eosin. We observed marked alveolar edema (arrow) and septal thickening (arrowhead) after the instillation of saline. Similar changes were found when GABA and bicuculline were instilled. Scale bar, 40 μm.

Histopathologic analysis was performed after injurious ventilation and instillation of GABA-receptor modulators. GABA reduced edema-fluid accumulation when compared with that in saline-instilled animals (Figure 2). Bicuculline effectively reduced GABA-mediated decrease in fluid accumulation, indicating that GABA_A _receptors were involved in this process. Histopathology also indicated significant septal thickening after high-volume ventilation. However, alveolar septa were normal after instillation of GABA. Instillation of bicuculline abrogated the GABA-mediated effect (Figure 2).

### Effect of GABA on apoptosis induced by high-tidal-volume ventilation

**Figure 3:**
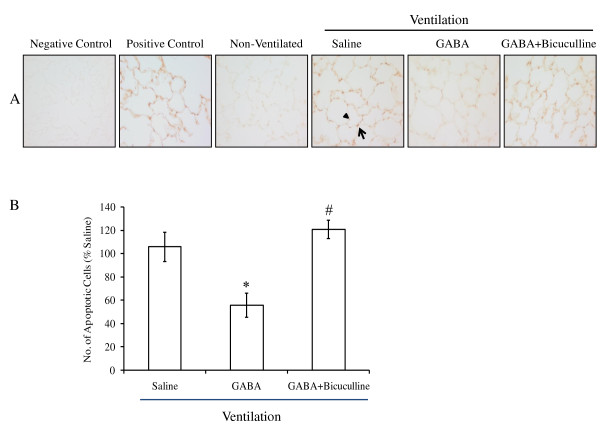
**Effect of γ-aminobutyric acid (GABA) on apoptosis in ventilator-induced lung injury**. The left lungs were isolated from rats after high-tidal-volume ventilation. The lung sections were processed for studying the changes in apoptosis by using terminal deoxynucleotidyl transferase dUTP nick-end labeling (TUNEL) staining. **(A) **Shown are the representative photomicrographs (×400) of lung tissues showing TUNEL-positive cells. Apoptosis was observed in AEC II (arrow) and AEC I (arrowhead) after instillation of saline. Scale bar, 40 μm. **(B) **The numbers of TUNEL-positive cells were counted in 15 randomly chosen fields. The results were expressed as a percentage of that in the control animal. The total number of TUNEL-positive cells was 51.0 ± 7.9 in saline-instilled animals. Shown are the means ± SEM (*n *= 6 to 8 per group). *P < 0.05 versus saline; ^#^*P *< 0.05 versus GABA.

TUNEL assay was performed on lung sections for studying apoptosis. High-tidal-volume ventilation resulted in apoptosis in epithelial cells, as indicated by TUNEL staining (Figure 3). Apoptosis was observed in AEC I and AEC II. The numbers of apoptotic cells were counted in 15 randomly chosen fields to quantify the changes (*n *= 6 to 8). The total number of TUNEL-positive cells was 51.0 ± 7.9 in saline-instilled animals. GABA reduced the number of apoptotic cells (22.9 ± 5.8). Bicuculline abolished the GABA effect (60.8 ± 1.8). Thus, protective effects of GABA might be due to reduced apoptosis of pulmonary epithelial cells.

### Effect of GABA-receptor modulators on ventilator-induced lung vascular damage

**Figure 4:**
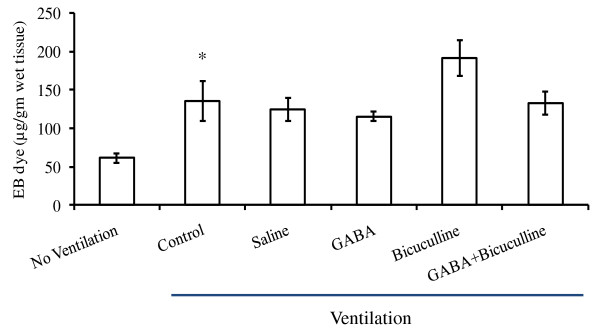
**Effect of γ-aminobutyric acid (GABA)-receptor modulators on vascular damage after high-tidal-volume ventilation**. Rats were anesthetized and injected with Evans Blue (EB) dye (20 mg/kg. BW) with and without the instillation of GABA (500 μ*M*) and/or bicuculline (200 μ*M*). The animals were ventilated first with a tidal volume of 8 ml/kg BW for 30 minutes. Later the tidal volume was increased to 40 ml/kg BW, and they were ventilated for 60 minutes. At the end of ventilation, the lungs were analyzed for EB-dye extravasation. Shown are the mean ± SEM (*n *= 4 to 6 per group). *P < 0.05 versus nonventilated animals.

To determine whether the protective effects of GABA were due to decreased pulmonary vascular damage, EB-dye extravasation was evaluated. No differences in EB-dye extravasation (micrograms per gram wet tissue) were found between those with instillation of saline and those without instillation (115.8 ± 15.23 versus 135.5 ± 25.7). GABA did not significantly affect extravasation (115.8 ± 5.5). The amount of EB dye in the lung when GABA and bicuculline were instilled was 135.8 ± 14.7 (Figure 4). Thus, no significant differences in pulmonary vascular damage were found after instillation of GABA-receptor modulators. The improvement in wet-to-dry ratio may not be due to differences in pulmonary vascular damage.

### Effect of GABA on alveolar fluid clearance

**Figure 5:**
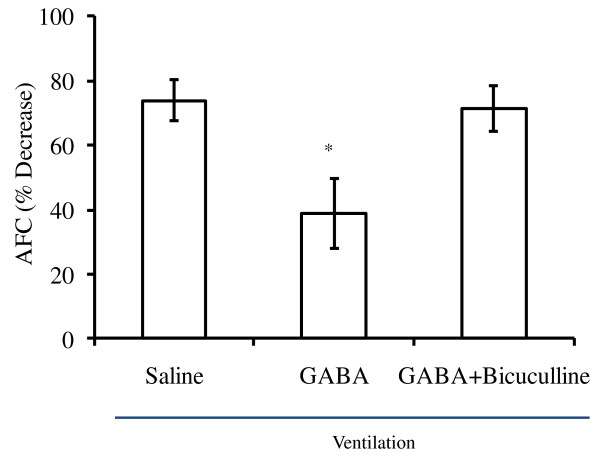
**Effect of γ-aminobutyric acid (GABA) on alveolar fluid clearance after ventilator-induced lung injury**. Adult female rats were subjected to high-tidal-volume (40 ml/kg BW) ventilation for 60 minutes with and without instillation of GABA (500 μ*M*) and/or bicuculline (200 μ*M*). A 5% bovine serum albumin along with 1 mg/ml FITC-albumin was included in the instillate for estimating alveolar fluid clearance. A decrease in FITC-albumin concentration was then calculated. Shown are the results of the percentage decrease in FITC-albumin concentration after ventilation for 1 hour. Shown are the mean ± SEM (*n *= 4 to 5 per group). *P < 0.05 versus saline.

We hypothesized that the decrease in wet-to-dry ratio might be due to increased AFC. To determine AFC, FITC-albumin (1 mg/ml) along with 5% unlabeled albumin was instilled into the left lungs, and changes in their concentrations were monitored after mechanical ventilation. High-tidal-volume ventilation decreased the FITC-albumin concentration by 75% in saline control animals, indicating decreased AFC (Figure 5). GABA decreased FITC-albumin concentration by 50% only. However, when bicuculline and GABA were instilled together, the decrease in FITC-albumin was similar to that observed in controls. Thus, bicuculline effectively inhibited the GABA-mediated effect on AFC.

## Discussion

Mechanical ventilators are increasingly used for life-saving procedures. Although they are indispensable, they cause lung injury, characterized by pulmonary edema. Modulating ion-channel activities can improve the outcomes of acute lung injury. This present study, for the first time, investigated the role of GABA receptors in modulating ventilator-induced lung injury. GABA decreased pulmonary edema, as shown by the decreased wet-to-dry ratio. The reduced pulmonary edema was due to the improved AFC and a decreased apoptosis.

Ventilator-induced lung injury is characterized by pulmonary edema and inflammation [27]. We observed increases in total BALF protein, wet-to-dry ratio, and vascular damage, as indicated by EB-dye extravasation. The conditions used to induce lung injury vary among investigators and laboratories. For example, tidal volumes range from 20 to 40 ml/kg BW, and ventilation times, from 40 minutes to a few hours. Sometimes, ventilation was done with 100% oxygen. Thus, a great variation is noted in the lung-injury parameters. Wet-to-dry ratio is the simplest method to use to evaluate pulmonary edema. However, it does not differentiate between interstitial and alveolar edema [1]. The wet-to-dry ratios in our study were similar to those in Sprague-Dawley rats [35,38,48], but higher than those reported in Wistar-Kyoto rats [49]. The increase in wet-to-dry ratio was evident as early as 30 minutes, but remained the same for up to 60 minutes. Thus, our lung-injury model conforms with those in earlier studies.

Histopathologic analysis indicates that alveolar epithelium was relatively less damaged after the instillation of GABA when compared with saline. Thus GABA might have protected alveolar epithelium from damage, leading to the alleviation of ventilator-induced lung injury. Indeed, GABA reduced apoptosis of distal lung epithelial cells. Increased apoptosis of distal lung epithelial cells occurs in acute lung injury (ALI) and acute respiratory distress syndrome (ARDS) in human patients [50]. Lung epithelial cell apoptosis is one of the critical parameters for evaluating lung injury [51]. Previous studies have shown that apoptotic inhibitors reduce endotoxin-mediated lung injury [52]. GABA attenuated ischemia-mediated neuronal apoptosis [53]. Mechanical ventilation has been shown to activate mitogen-activated protein kinase, which in turn activates various apoptotic pathways [23,24,54]. We think that the GABA-mediated reduction in apoptosis enables the distal lung epithelial cells to function properly, including AFC.

Mechanical ventilation for 1 hour decreased AFC in adult rats. The reduction in AFC by mechanical ventilation in our studies was smaller than that in earlier studies in rats [30,48,55]. The differences in AFC rates could be due to the systems used: *in situ *and isolated perfused lungs versus *in vivo *in our case. AFC was measured in the absence of blood flow in the perfused-lung models. Our current data showed that GABA prevented the pulmonary edema. Our data further support that the effects of GABA on the ventilator-induced pulmonary edema are due to, at least in part, an increase in fluid clearance. High-volume ventilation causes vascular damage [56] and increases permeability to proteins and small solutes [30,55,57]. However, instillation of GABA did not prevent the vascular damage. Furthermore, GABA increased AFC, as directly measured by the FITC-albumin dye. Previous studies have reported that β-adrenergic agonists increase AFC in the injured lungs, and this effect is effectively blocked by amiloride [30,55], suggesting a role of ENaC in the resolution of edema.

In the present study, GABA increased AFC in ventilator-induced lung injury. We think GABA-mediated effects are due to its effects on the increased Cl^- ^influx and not efflux. In normal adult rats, GABA receptors mediate Cl^- ^effluxes and decrease AFC [11]. Two possible explanations exist:

1. It is possible that GABA mediates Cl^- ^influxes in the lung under injurious conditions. GABA receptors have been shown to switch the Cl^- ^conductance patterns in fetal and adult cells, depending on intracellular Cl^- ^concentrations [14,16,58].

2. Our recent study showed that AEC I and AEC II operate Cl^- ^fluxes in completely opposite directions, even when activated by the same ligand, UTP. AEC II mediates Cl^- ^efflux, whereas AEC I mediates Cl^- ^influx [15]. Mechanical ventilation might injure AEC I, leading to cessation or inhibition of Cl^- ^influxes. GABA prevents the death of AEC I and thus an increased of net influx. It is unknown whether AEC II reverses Cl^- ^fluxes in injurious conditions.

## Conclusions

Our results, for the first time, reveal that GABA exerts protective effects on ventilator-induced lung injury. The GABA-mediated effect is due to increased AFC and reduced apoptosis of epithelial cells. Thus, GABA might be of use in clinical conditions to reduce ventilator-induced lung injury.

## Key messages

• GABA reduced ventilator-induced pulmonary edema through its effects on alveolar fluid clearance and apoptosis.

## Abbreviations

AEC I: alveolar epithelial cell type I; AEC II: alveolar epithelial cell type II; ALI: acute lung injury; AFC: alveolar-fluid clearance; ATP: adenosine triphosphate; BAL: bronchoalveolar lavage; BALF: bronchoalveolar lavage fluid; BW: body weight; CFTR: cystic fibrosis transmembrane conductance regulator; D1: dopamine type 1 receptor; EB: Evans blue; ENaC: epithelial Na^+ ^channels; FiO_2_: fraction of inspired oxygen in a gas mixture; GABA: γ-aminobutyric acid; GABA_A_: γ-aminobutyric acid type A; GABA_A_-ρ: γ-aminobutyric acid type A composed of ρ (rho) subunits; KCC: K^+^-Cl^- ^cotransporter; NKCC: Na^+^-K^+^-2Cl^- ^cotransporter; NSC: cation-nonselective channel; PEEP: positive end-expiratory pressure; TUNEL: terminal deoxynucleotidyl transferase dUTP nick-end labeling; UTP: uridine triphosphate.

## Competing interests

The authors declare that they have no competing interests.

## Authors' contributions

NRC carried out all of the experiments and drafted the manuscript. LL conceived of the study, participated in its design and coordination, and helped to draft the manuscript. Both authors read and approved the final manuscript.
